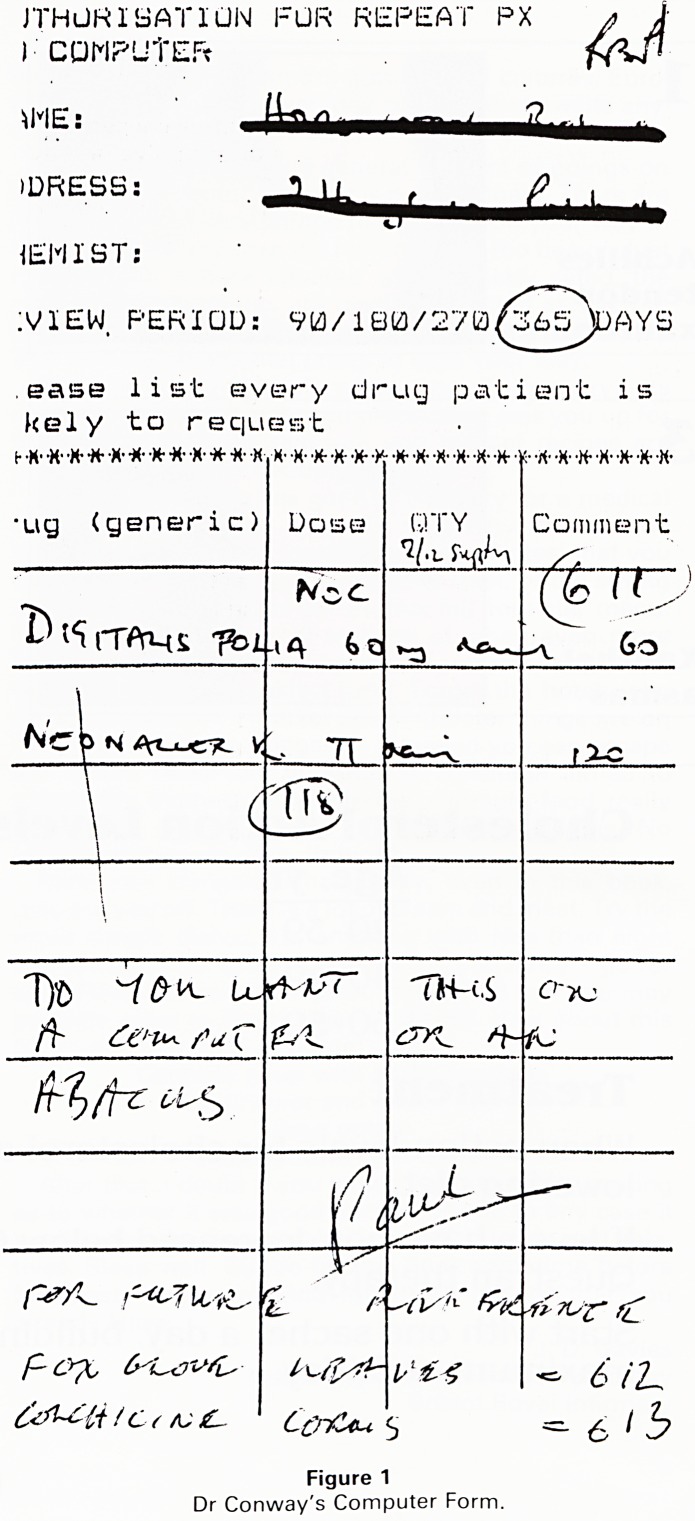# Letters to the Editor

**Published:** 1986-04

**Authors:** Roger B. Pardoe


					Sir,
The October issue of the Journal with its three articles on
Computers in General Practice was of great interest. The
most important factor in computerisation in General
Practice is the necessity of having at least one member of
the team who equips himself with the basic knowledge
and has the enthusiasm to persuade his Partners that it is
a worthwhile project. After all, they have to pay.
In my Practice it is Dr. Conway who has shown this
enthusiasm and all information is fed to the computer by
means of this form (1). A computer is only as good as the
programmer and he occassionally, poor chap, hiccups
over the information sent to him. So, when his obviously
'out of the ark' Partner sent him this slip, he felt that it
should not be put in the basket but returned to sender
with this apt comment.
May I say in my defence as the instigator of this drug
treatment that the patient had been seen by many Con-
sultants, all of whom have agreed that she needs Digita-
lis, but none have been able to persuade her to take a
more modern form?they all make her sick. Perhaps the
art of medicine is not yet dead.
Koger b. Pardoe
JTHURIBAT1UN FUR REPEAT FX
) -'COMPUTER
f&J
Im
J2.
Aj.
WE:
(DRESS:
DEMIST:
;VIEW. PERIOD: 90/ 180/270^365^0AYS
ease list every drug patient is
kely to request
(? K' * * * * * * * * K .K- M- X- K- * -K-
?uy (generic) Dose
D
N'cp N fi(UjLJcrr^
(35
1> u.|
6t' u^ /Vc
/^/v_
f~ OJC
{C< flj ?-
Ncc.
JL
i/7
CtfiCiu 5
* ?#? -X'-H-'K'-K-
QTY
it- -K- -K- -K- * * ???
Comment
/tH^
'7
L
? rr
Go
C'-AJ
6 i*L
6 1 b
Figure 1
Dr Conway's Computer Form.
46

				

## Figures and Tables

**Figure 1 f1:**